# Successful Renal Replacement Therapy for a Patient with Severe Hemophilia after Surgical Treatment of Intracranial Hemorrhage and Hydrocephalus

**DOI:** 10.1155/2011/824709

**Published:** 2011-11-17

**Authors:** Noriko Kato, Masami Chin-Kanasaki, Yuki Tanaka, Mako Yasuda, Yukiyo Yokomaku, Masayoshi Sakaguchi, Keiji Isshiki, Shin-ichi Araki, Shigeru Ohta, Takashi Uzu

**Affiliations:** ^1^Department of Medicine, Shiga University of Medical Science, Seta Tsukinowa-cho, Otsu, Shiga 520-2192, Japan; ^2^Clinical Medical Education Center for Physicians, Shiga University of Medical Science, Seta Tsukinowa-cho, Otsu, Shiga 520-2192, Japan

## Abstract

A 21-year-old Japanese male with severe hemophilia A was developed end-stage renal failure. He was placed on combination therapy with peritoneal dialysis (PD) and hemodialysis (HD). Eight months later, he developed a hypertensive cerebral hemorrhage. After emergency surgery, he was managed with PD without HD to avoid cerebral edema. One month later, his renal replacement therapy was switched to HD (three times a week) from PD, since a ventriculoperitoneal shunt catheter was placed to treat his hydrocephalus. HD could be performed safety without anticoagulant agents on condition that factor VIII is given after every HD.

## 1. Introduction

Hemophilia is a hereditary X-linked recessive hemorrhagic disease and occurs in about 1 out of 10,000 and affects an estimated 400,000 people globally [1]. The severity level of hemophilia A was categorized as severe if the activity was <1%, whereas 1 to 5 percent and >5 percent of normal are defined as moderate and mild disease, respectively [2]. Recently, the prevalence of end-stage renal disease is increasing in patients with hemophilia, because they are surviving into their 60s and beyond [3, 4]. Concerning the renal replacement therapy for hemophilia patients, it is important that the bleeding risks should be minimized. Peritoneal dialysis (PD) has been recommended as the renal replacement therapy for the hemophilia patients with end-stage renal disease, since hemodialysis (HD) patients have high risks of hemorrhage and hematoma formation with repeated access of an arteriovenous fistula [5]. However, patients with extensive abdominal adhesion, psycho-neurological problems, abdominal mechanical problems (e.g., hernia sack, subcutaneous leak, or ventricular-peritoneal shunt) were not suitable for PD. This paper presents a case of a young male with severe hemophilia received PD and HD according to his condition. 

## 2. Case Report

**Figure 1:**
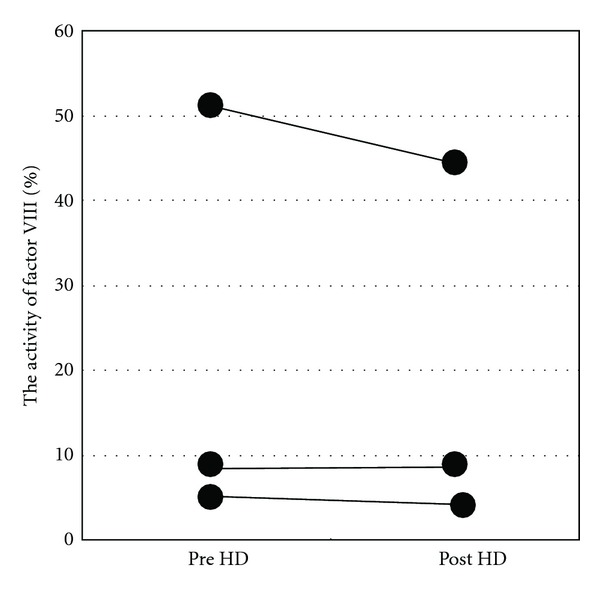
Factor VIII activity before and after hemodialysis sessions.

**Figure 2:**
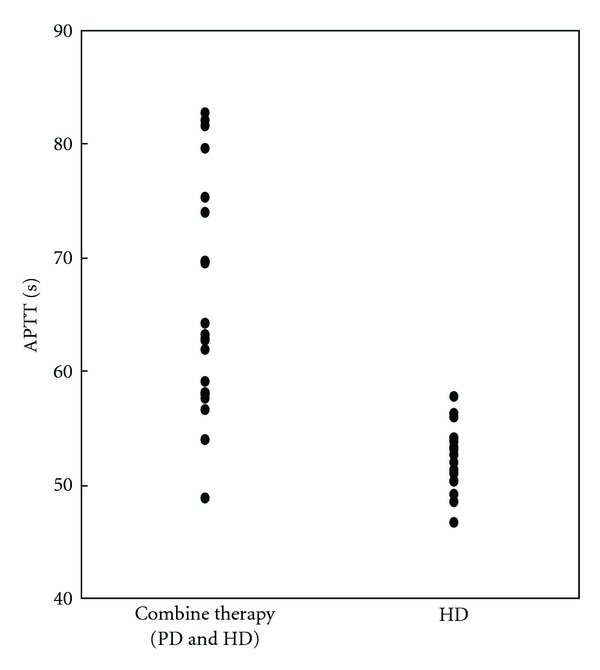
The activated partial thromboplastin time during combined therapy with peritoneal dialysis (PD) and hemodialysis (HD) or HD. (65.9 ± 9.9 versus 52.3 ± 2.9, *P* < 0.01). APTT: activated partial thromboplastin time.

A Japanese male who was diagnosed as having severe hemophilia A (factor VIII activity less than 1%) due to a large thigh hematoma at 10-month old. He was diagnosed with chronic kidney disease at age of 18 on the basis of persistent proteinuria with hematuria. He was diagnosed with congestive heart failure and end-stage renal disease at age of 21. He started to undergo PD, but heart failure recurred one month later because of his poor self-management. Thereafter, he underwent combination therapy with HD (for 4 hours, once per week) and PD (1.5% Dianeal PD-2, 4 times per day, 6 days per week). Factor VIII (1000 U) was administered about three times a week (once a week after HD and irregular self-injection). He developed suddenly severe headache eight months after the beginning of the combination therapy. When he presented at the emergency room, his blood pressure was 200/100 mmHg. His blood tests showed prolonged APTT (110 second). In spite of immediate treatment by antihypertentsive medication (oral nifedipine), one hour later, he had a generalized convulsion due to intracerebral hemorrhage. The next day, his cerebral hemorrhage enlarged and his consciousness level worsened despite administration of factor VIII and intravenous anti-hypertensive medications. Emergency surgery was performed to remove the hematoma. He received a large amount of factor VIII to achieve an APTT level less than 40 seconds for one week. He regained consciousness after surgery and was managed with PD without HD to avoid cerebral edema. One month later, his renal replacement therapy was switched to HD (three times a week) from PD, since a ventriculoperitoneal shunt catheter was placed to treat his hydrocephalus. Neither clot formation nor hemorrhage was found after hemodialysis which was performed without anticoagulant agents. He received 1000 U of factor VIII before removal of the dialysis needle at the end of every HD session to maintain the APTT level between 50 sec and 60 sec. The factor VIII levels were not influenced by the hemodialysis (Figure 1), indicating that factor VIII did not pass through the dialysis filter.The APTT levels under management with HD were significantly shorter than those under combination therapy (PD and HD) (Figure 2). He could be discharged from hospital, and he underwent maintenance dialysis safely without anti-coagulant as outpatient thrice weekly.

## 3. Discussion

There are some reports about appropriate anti-coagulation during hemodialysis processes in patients with hemophilia [6, 7]. A combination regional heparinization and factor VIII before and after dialysis has been recommended to prevent clot formation in the extracorporeal circulation as well as minimize bleeding. However, Lambing et al. reported a patient with hemophilia who was treated with chronic HD without anticoagulation [8]. The current case also could maintain HD safety without anti-coagulation under administration of factor VIII at the end of every HD. Figure 1 revealed that the postdialysis activity of factor VIII was similar to the predialysis activity in this case. Since the molecular weight of factor VIII (light chain 80,000 Da and heavy chain 90,000–200,000 Da) is greater than that of albumin (66,300–69,000 Da), factor VIII would not pass through the dialysis filter.

Prophylactic administration of clotting factor concentrates for hemophilia patients is advisable prior to engaging in activities with higher risk of injury to prevent bleeding. Currently, the most commonly suggested protocol for prophylaxis is the infusion of 25–40 IU/kg of clotting factor concentrates three times a week for those with hemophilia A [2, 9]. The current patient needed prophylactic administration of factor VIII due to severe hemophilia A. Treatment with 1000 U of factor VIII every HD (three times a week) for him maintained the activated partial thromboplastin time (APTT) between 50 and 60%. Regular prophylactic administration after HD could achieve more stable anti-coagulation level than irregular self-injection which was done under combination therapy (Figure 2). Factor VIII might leak in peritoneal fluid.

The incidence of intracerebral hemorrhage in dialysis patients is reported to be more than 5 times higher than that in the general population [10]. Therefore, cerebral hemorrhage is a risky complication in patients with hemophilia receiving dialysis therapy. The current case developed severe cerebral hemorrhage in spite of intensive care for hypertension and hemophilia. His cerebral bleeding might be caused by poorly controlled hypertension and severe coagulation disorder. This case suggests that blood pressure should be strictly controlled in hemophilia patients, especially those treated with renal replacement therapy.
